# Books and Authors

**Published:** 1912-04

**Authors:** 


### BOOKS AND AUTHORS

**Honan's Handbook to Medical Europe. Dr. James Henry Honan,** Chicago. Published by P. Blakiston's Son & Co., Philadelphia. $1.50.

This little book begins by giving advice to those visiting Europe for the first time. Letters to foreign professors except those from warm personal friends are considered useless. Each country is then considered separately, its advantages and medical laws discussed. The countries are further subdivided into cities, under which head is given a list of the hospitals and staff together with the courses given by each and fees charged. Careful perusal of this book must prove of inestimable value to those contemplating a European trip.

---

**Nervous and Mental Diseases.** By **Archibald Church,** M. D., Professor of Nervous and Mental Diseases and Medical Jurisprudence in Northwestern University Medical School, Chicago; and **Frederick Peterson,** M.D., Professor of Psychiatry, Columbia University. Seventh edition, revised. Octavo volume of 932 pages, with 338 illustrations. Philadelphia and London: W. B. Saunders Company, 1911. Cloth, $5.00 net; half morocco, $6.50 net.

It is with considerable personal gratification that many of our readers will peruse this book. Dr. Peterson was a graduate of the University of Buffalo in 1879 and began the practice of medicine in Buffalo. Progressiveness has characterized his work from the time that he scandalized some of the older physicians in a local hospital by measuring intestines at necropsy in centimeters instead of inches. It is obviously impossible to do justice to such a work in a brief review. Perhaps the best index to its thoroughness, conciseness in spite of its large size, and comprehensiveness is the fact that only 29 pages are given to hysteria. When we consider how easy it is to pad a work on this subject, by interminable descriptions and discussions of hysteria, we comprehend that this work is almost encyclopaedic.

---

**Blair's Pocket Therapeutics.** By **Thomas S. Blair,** Harrisburg. Published by The Medical Council Co., Philadelphia, 1911. 370 pages, $1.00.

This is a pocket condensation both of medical practice and therapeutics, containng an astonishing amount of information of great utility, especially for the recent graduate liable to encounter problems at a distance from counsel or a library. It is easy to sneer at such compends as superficial but they save lives.

---

**Studies of the Niagara Frontier, Frank H. Severance,** Vol. 15 of the Buffalo Historical Society Proceedings, 1911. 437 pages, 3 maps, cloth.

Seventeen eighteenth century and a few earlier descriptions of the Niagara Frontier, and a charming chapter devoted to "History that isn't so" are perhaps the most readable but the main value of the work rests with its exhaustive study of the history and literature dealing with the region. The editor has long been the acknowledged authority in this field and it is fortunate that his years of study are to be permanently preserved.

---

**Diseases of the Skin and the Eruptive Fevers.** By **Jay Frank Schamberg,** M.D., Professor of Dermatology and Infectious Eruptive Diseases in the Philadelphia Polyclinic and College for Graduates in Medicine. Second edition, revised. Octavo of 573 pages, 235 illustrations. Philadelphia and London: W. B. Saunders Company, 1911. Cloth, $3.00 net.

The frequent use of tables, as well as the abundance of illustrations, facilitates the acquisition on information from this book. The final chapter on the eruptions occurring occasionally but not typically in the course of various fevers, not classified as eruptive, in serum reactions, etc., is especially valuable because it has rendered available a great mass of scattered observations.

---

**Health and Medical Inspection of School Children. Walter S. Cornell,** M.D., Philadelphia. Published by the F. A. Davis Co., Philadelphia, 1912. 614 pages, 200 illustrations, $3.00.

This is a timely book, presenting in concise form the varied problems which many cities are just beginning to meet. In the absence of established precedent and long experience, Dr. Cornell has established a model which will probably be copied by other authors soon. His book should be in the hands of every school principal and superintendent, every school inspector or aspirant for such a position. More than this, the description of physical defects actually encountered in children, the demonstration of hereditary taints, the careful study of feeding, and many other details render the book valuable to the practitioner. We wish, too, that it might be purchased at public expense and placed in the hands of state and municipal legislators.

---

**Practical Gynecology, E. E. Montgomery,** M.D., LL.D., Philadelphia; P. Blakiston's Son & Co., Philadelphia, 1912. 4th edition, 854 pages, 589 illustrations, $6.00.

Nearly twenty-five years ago, as a post-graduate student, we had the benefit of Dr. Montgomery's clinics at Blockley Hospital (locally known as the A'ms House) and probably saw one or two of the identical anomalous cases described in his book. It has been a pleasure to watch his development. His book has been brought nearer perfection with each edition and is a masterly treatise. To pick a minor flaw, we are a little disappointed that so scholarly a gentleman should retain without apology, such hybrid words as vaginitis, vulvitis, etc.

---

**Progressive Medicine,** Vol. 14, No. 1, March, 1912. Edited by Hobart **A. Hare** and **Leighton F. Appleman.** Published by Lea & Febiger, Philadelphia. This volume contains a resume of Surgery, Infectious Diseases, Pediatrics, Laryngology, Otology, under competent department editors. 376 pages. Quarterly, $6.00 per annum.

This is a most valuable digest of current literature, intermediate between the magazine and the text book. It is, we think, too valuable for a paper cover.

**Transactions of the Thirty-third Annual Meeting of the American Laryngological Association.** Held at Philadelphia, Pa., in May 1911. Published by the association.

These Transactions are published for the members of the Association, but a few copies are available for outside sale. The volume contains the complete records of the meeting, including all papers read, the business transacted and a list of the members of the Association. Although the papers were written by specialists for specialists, a number of them are of great general interest, and the general practitioner would be benefited by their perusal. Those papers are: The President's address with its suggestions upon the relationship between diseases of the nose and throat and general conditions; the paper upon Epiglottidectomy, by Dr. L. B. Lockhard of Denver, in which he tells of the relief of pain after removal of the epiglottis in tuberculosis of that organ; that on Syphilis of the Larynx, by Dr. G. C. Stout, of Philadelphia, with its valuable hints on treatment, both local and general; the paper on the Relation of Enlarged Tonsils to Enudocarditis, by Dr. A. C. Getchell, of Worcester, Mass., with its thought as to the origin of rheumatism; and that with the title, Are the Tonsils a Menace or a Protection? by Dr. H. L. Swain, of New Haven, Conn.

The remaining papers, as well as those quoted, are of the high order of merit usual in the papers presented before this Association. They are scholarly and of very deep scientific interest. Anyone choosing to read this book will not waste his time.

---

**The Taylor Pocket Case Record.** By **J. J. Taylor,** M. D. Published by the Medical Council Co., Philadelphia, 1911. $1.50.

This is not a visiting list but a series of blank forms bound together, with alphabetic index. Two pages, facing each other, are allowed for a case, with spaces for listing in the usual form, noting principal symptoms, findings, diagnosis and treatment, with record of subsequent attendance. While obviously not adapted to keeping elaborate records, it is a good working memorandum book for the general practitioner and it can be used in the office and in a round of calls. One minor fault should be and undoubtedly will be remedied—the paper is so thin that one is liable to be confused by seeing at one time the writing on both sides of one leaf and on one side of the next leaf.

**Digest of Comments on the Pharmacopoeia of the U. S. A.,** and on the **National Formulary**, for the Calendar Year 1909, by **Murray Galt Motter** and **Martin I. Wilbert,** No. 79 of the Hygienic Laboratory. Government Printing Office. 735 pages.

The large number of articles abstracted requires great brevity and, in many instances, one has mere opinions to deal with. These criticisms should certainly be carefully studied by the committees in charge of the Pharmacopoeia, National Formulary, etc. There are also many references to strictly analytic methods, as of the examinations of blood, urine, faeces, etc., too brief to be of value except as a bibliographic list.

---

Vol. VI, **Transactions of The American Association of Genito-Urinary Surgeons,** maintains the same standard of excellence as its predecessors.

Ruggels, Diseases of the Verumontanum, states that nitrate of silver applications to the diseased organ must consist of strong solutions or even the solid pencil to be effectual.

Swinburne, describes a new Complement Fixation Test for gonorrhoea similar to the Wassermann reaction. The objection to this test is the uncertainty when positive as to whether the patient is still infectious or whether antibodies persist after recovery.

Binney, advises that a mercury manometer be connected to a catheter while the urine is flowing out as a test for bladder atony.

Cabot, presents a simple scale for the phenosulphonephthalein test or renal function. Ten tests tubes are filled with solutions of the drug varying in strength from 5 to 50 per cent. alkalinized and sealed with paraffin. The urine to be tested is diluted to 1000 c.c. some is then poured into a test tube of similar size and compared with the scale. By this method the solution may be estimated within 2 per cent. He considers this test to be simpler in technic and more accurate in its results than any other.

